# Evaluation of EMG, force and joystick as control interfaces for active arm supports

**DOI:** 10.1186/1743-0003-11-68

**Published:** 2014-04-19

**Authors:** Joan Lobo-Prat, Arvid QL Keemink, Arno HA Stienen, Alfred C Schouten, Peter H Veltink4, Bart FJM Koopman

**Affiliations:** 1 Department of Biomechanical Engineering, University of Twente, Drienerlolaan 5, 7522 NB Enschede, The Netherlands; 2 Department of Physical Therapy and Human Movement Sciences, Northwestern University, 645 N Michigan Ave Suite 1100, 60611 Chicago, IL, USA; 3 Department of Biomechanical Engineering, Delft University of Technology, Mekelweg 2, 2628 CD Delft, The Netherlands; 4 Department of Biomedical Signals and Systems, University of Twente, Drienerlolaan 5, 7500 AE Enschede, The Netherlands

**Keywords:** Control interface, Electromyography, Force, Joystick, Performance evaluation, Learning curve, Human-operator

## Abstract

**Background:**

The performance capabilities and limitations of control interfaces for the operation of active movement-assistive devices remain unclear. Selecting an optimal interface for an application requires a thorough understanding of the performance of multiple control interfaces.

**Methods:**

In this study the performance of EMG-, force- and joystick-based control interfaces were assessed in healthy volunteers with a screen-based one-dimensional position-tracking task. The participants had to track a target that was moving according to a multisine signal with a bandwidth of 3 Hz. The velocity of the cursor was proportional to the interface signal. The performance of the control interfaces were evaluated in terms of tracking error, gain margin crossover frequency, information transmission rate and effort.

**Results:**

None of the evaluated interfaces was superior in all four performance descriptors. The EMG-based interface was superior in tracking error and gain margin crossover frequency compared to the force- and the joystick-based interfaces. The force-based interface provided higher information transmission rate and lower effort than the EMG-based interface. The joystick-based interface did not present any significant difference with the force-based interface for any of the four performance descriptors. We found that significant differences in terms of tracking error and information transmission rate were present beyond 0.9 and 1.4 Hz respectively.

**Conclusions:**

Despite the fact that the EMG-based interface is far from the natural way of interacting with the environment, while the force-based interface is closer, the EMG-based interface presented very similar and for some descriptors even a better performance than the force-based interface for frequencies below 1.4 Hz. The classical joystick presented a similar performance to the force-based interface and holds the advantage of being a well established interface for the control of many assistive devices. From these findings we concluded that all the control interfaces considered in this study can be regarded as a candidate interface for the control of an active arm support.

## Background

Several active arm supports are currently available and used to increase the independence and the quality of life for patients suffering from neuromusculoskeletal disorders [1,2]. The operation of these active devices is mediated by a control interface that detects the user’s movement intention. The design of the control interface in response to specific user needs and capabilities is crucial for the usability and success of the device.

Electromyography-based interfaces are the most common method used for the control of active prostheses and orthoses [3-7]. Myoelectric prostheses are controlled by measuring electromyographic (EMG) signals from two independent residual muscles or by distinguishing different activation levels of one residual muscle. Switching techniques such as muscle co-contraction or the use of mechanical switches or force-sensitive resistors are implemented for the sequential operation of several degrees of freedom (DOF) [8]. In the case of active orthoses, these are controlled by estimating the muscular joint torques from the EMG signals of the muscles that mainly contribute to the supported motion [3,4,7]. Recently, innovative pattern recognition algorithms [5] and surgical procedures such as targeted muscle reinnervation [9] are being developed in order to improve the functionality of EMG-based interfaces.

Force-based interfaces have been used in assisted-powered wheelchairs [10] where the wheelchair detects and amplifies the force applied by the user. Recent studies implemented six-DOFs force-torque sensors [11,12], or simple force sensor resistors for the control of active upper-extremity orthoses [13] and prosthesis [14]. These kind of interfaces generally implement admittance control strategies where the output acceleration, velocity or position is related to the input force [15]. Haptic force-based control interfaces are very often implemented in rehabilitation robots where patients need to train to regain control, mobility and strength [16,17].

Joysticks have been used for the control of powered wheelchairs [18] and external robotic arms [19,20]. Recent studies also investigated the performance of controlling prosthetic arms with the residual shoulder motion measured with a two-DOF joystick [14,21]. Furthermore, Johnson et al. [22] developed a five-DOF upper-extremity orthoses, in which the position of the end point was controlled with a joystick operated by the contralateral hand.

While there is a large variety of control interfaces, only few studies have focused on their formal performance evaluation and comparison [23-25]. As a consequence, there is a lack of knowledge as to which one is the most suitable for a specific type of impairment and task. Currently, there is no basic consensus on how to evaluate the performance of control interfaces, which prevents their objective evaluation and comparison.

The selection of the most suited control interface for a specific application requires a better understanding of the limitations and capabilities of the different control strategies, through objective and quantitative evaluations during functional tasks. One example of this approach is the study by Corbett et al. [23], which compared wrist control of angle, force, and EMG as interfaces for upper-extremity prosthesis during a one-dimensional position-tracking task. The control interfaces were evaluated at 1 Hz, which according to the authors it is a tracking frequency optimal for direct-position control. The results of the study showed that EMG and force interfaces did not outperform their golden standard angle-based interface in all the performance descriptors considered (tracking error, bandwidth and information transmission rate). But they did show that EMG was significantly better than force in terms of tracking error.

While wrist control is appropriate to evaluate interfaces for the operation of active hand prostheses, the control of active arm supports is preferably achieved by interfacing with signals from more proximal joints. Therefore, our ultimate interest in developing active arm supports for individuals with muscular weakness required extending the aforementioned work [23] with a comparative study of the performance, learning characteristics and subjective preference of control interfaces that used signals from either elbow or shoulder joints. Additionally, we were interested in evaluating the control interface performance for a bandwidth beyond 1 Hz in order to capture the limitations of the human-operator.

Here we report tests performed by eight healthy subjects using a screen-based one-dimensional position-tracking task. Healthy individuals were chosen to provide a baseline performance measure and to serve as a reference on the potential value of the control interfaces for active arm supports.

## Methods

We compared control interfaces based on physiological signals from the elbow muscles -EMG and force- because they are intrinsically related to the arm movement, and added a joystick interface as an alternative system that is more familiar to patients. The selected physiological signals were EMG signals from the muscles that mainly contribute to elbow flexion-extension and the force signals resulting from the activation of elbow flexion-extension muscles. Signals from the elbow muscles were preferred over those at the shoulder as they are easier to access with surface EMG.

Our motivation to test a classic hand-joystick is based on the fact that this type of interface is commonly used by individuals with severe muscular weakness to control electric wheelchairs, domestic devices and external robotic arms. Therefore, it makes sense to consider the option of also using this control interface to operate an active arm support. Comparing a classic hand-joystick to new interfaces (from a patient’s point of view) is especially relevant for the targeted patient group, as the performance of a new control interface needs to represent a meaningful improvement and worth the effort of learning and adapting to it.

The performance of each control interface was evaluated in terms of tracking error, gain margin crossover frequency, information transmission rate and effort. The learning characteristics were evaluated by analyzing the tracking error along a series of training trials. A model of the human-interface system was fitted to its estimated frequency response function (FRF) to evaluate the delay and gain parameters of each control interface. Finally, the eight participants were asked to list the control interfaces in order of preference.

The experimental procedure was approved by the medical ethical committee in the Arnhem-Nijmegen region (the Netherlands).

### Participants

A total of eight healthy males aged between 22 to 29 years participated in this study. All participants gave written informed consent, were right-arm dominant and had no experience with EMG- or force-based control interfaces. The experimental protocol was in accordance with the Research Ethics Guidelines of the Department of Biomechanical Engineering of the University of Twente (Enschede, The Netherlands).

### Experimental setup and protocol

**Figure 1:**
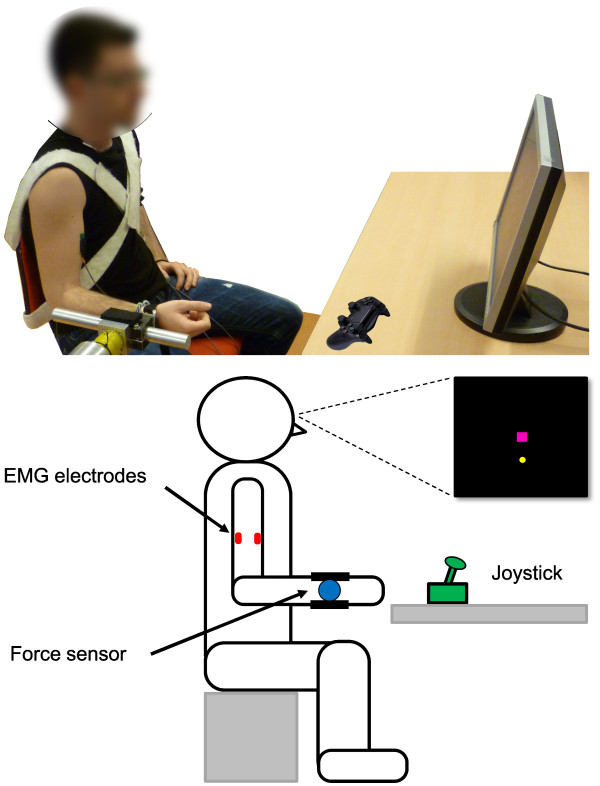
**Experimental setup.** Top) Picture of the experimental setup. Bottom) Schematic diagram of the experimental setup. The forearm of the participants was securely strapped to a rigid structure using a padded brace around the styloid processes. The EMG electrodes were placed at the biceps and triceps muscles. The resulting forces from the biceps and triceps activation where measured with a 1DOF force sensor located at the wrist. The joystick was located in front of the subject.

**Figure 2:**
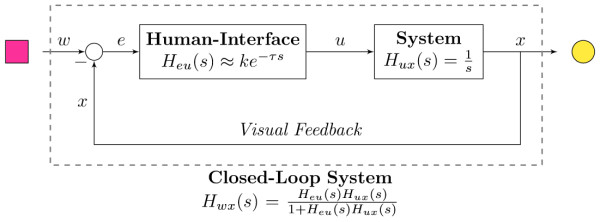
**Block Diagram of the position-tracking task.** The subject visually perceived the target (*w*) and cursor (*x*) positions. In order to minimize the error (*e*) between them, the human generated a control signal (*u*), using one of the control interfaces, which was mapped to the velocity of the cursor and subsequently integrated to obtain the cursor position.

**Figure 3:**
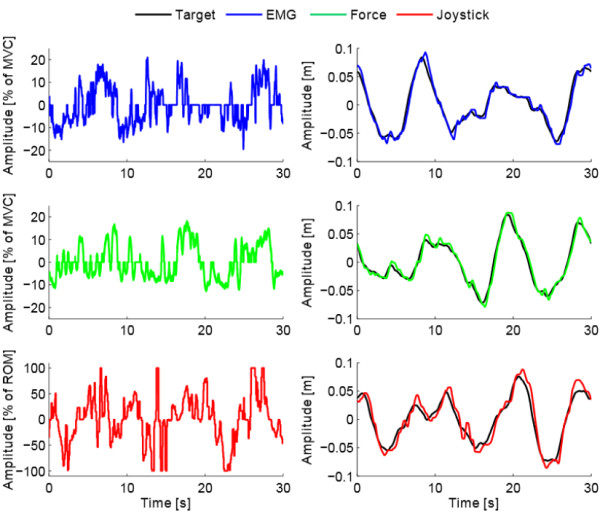
**Interface, target and cursor signals.** Left) EMG (blue), force (green) and joystick (red) signals measured by the control interfaces. The interface signals, which are proportional to the velocity of the cursor, were generated by one of the participants attempting to track the target. Right) Target and cursor position signals for each control interface resulting from the interface signals shown in the left part of the figure.

A one-dimensional position-tracking task was presented to the subjects on a computer screen by means of a C ^*#*^ (Microsoft Visual Studio, Microsoft Corporation, USA) graphical user interface. The subjects remained in a sitting position during all the experiment with the arm immobilized as shown in Figure 1. With the elbow flexed at 90 degrees, the forearm was securely strapped to a rigid structure using a padded brace around the styloid processes. During the experiment, the participants were asked to keep the cursor (yellow circle in Figure 1 and 2) as close as possible to the center of a dynamic target (magenta square in Figures 1 and 2), which moved according to a predefined multi-sine signal with a flat velocity spectrum (i.e. all frequency components of the target velocity had the same amplitude). The experimental task is represented in a block diagram form in Figure 2. The participant visually perceived the target (*w*) and cursor (*x*) positions, and in order to minimize the error (*e*) between them, the participant generated a control signal (*u*) using one of the interfaces (i.e. EMG, force or joystick), which was mapped to the velocity of the cursor and subsequently integrated to obtain the cursor position. Figure 3 shows a sample of the target and cursor positions and the corresponding control signals for each control interface.

The participants were asked to execute the tracking task with the three different control interfaces. The order in which the subjects tested each interface was randomized. For each interface, 10 training trials of 30 seconds and 3 evaluation trials of 180 seconds were performed. Training trials allowed the subjects to become familiar with the control interface and to get as close to their maximum performance as possible before starting the evaluation trials. The experimenter informed the participants after each training trial about the tracking error and encouraged him/her to improve it.

### Signal acquisition and conditioning

The 30 seconds position signal of the moving target (*x*) was generated from 10 sinusoidal signals with (i) logarithmically distributed frequencies between 0.1 and 3 Hz; (ii) amplitudes inversely proportional to frequency; (iii) and randomly assigned phases for each trial. The design of the input signal was adapted from the classical work of McRuer [26] who did extensive research on the modeling of human-machine systems.

The isometric EMG signals were measured from the biceps and the triceps brachii, where the activation of the biceps moved the cursor up and the activation of the triceps moved the cursor down. Two 99.9% Ag parallel bars (contact: 10 mm × 1 mm each) spaced 1 cm apart (Bagnoli DE-2.1. Delsys; Boston, Massachusetts) were placed in parallel with the muscle fibers according to the SENIAM (Surface ElectroMyoGraphy for the Non-Invasive Assessment of Muscles) recommendations [27]. The signals were amplified with a Delsys Bagnoli-16 Main Amplifier and Conditioning Unit (Delsys; Boston, Massachusetts) with a bandwidth of 20 to 450 Hz and a gain of 1000.

Forces resulting mainly from elbow flexion-extension muscles were measured at the forearm, using a custom made one DOF load cell attached between the padded brace and the ground. During the training trials subjects were instructed to use biceps and triceps muscles, avoiding the generation of force from shoulder or trunk movements. A force upwards (elbow flexion) moved the cursor up and a force downwards (elbow extension) moved the cursor down. For each subject, the offset force resulting from the weight of the arm was corrected at the beginning of the experiment.

Both the EMG and force signals were sent to a real-time computer (xPC Target 5.1, The MathWorks Inc; Natick, Massachusetts) by means of a National Instruments card (PCI-6229; Austin, Texas), which performed the analog-to-digital conversion with a sampling frequency of 1 KHz and 16-bits resolution. The controller was also running in the real-time computer and was connected through a local area network using TCP/IP protocol to a computer with Windows operating system (Microsoft Corporation, USA) which was displaying the tracking task by means of the C ^*#*^ graphical user interface.

For the joystick-based control interface we used the joystick of the PlayStation 3 controller (Sony Computer Entertainment; Miniato, Tokyo, Japan) which presents a similar range of motion than the joysticks used to control electric wheelchairs. A forward tilt of the joystick moved the cursor up and a backward tilt of the joystick moved the cursor down. The digital signal was sent to the real-time computer by means of a USB interface.

### Signal processing and normalization

In order to obtain the envelopes, the EMG signals were full-wave rectified and smoothed using a second order low-pass Butterworth filter with a cutoff frequency of 5 Hz as in [23,28]. Preliminary analysis revealed that a cutoff frequency of 5 Hz represents a good tradeoff between noise removal and control bandwidth. No filter was applied to the force and joystick.

Before starting the tracking task, subjects were asked to perform three maximal voluntary contractions (MVC) of three seconds for both biceps and triceps muscles. Both EMG and force signals were measured simultaneously during the MVCs and used to normalize the EMG and force signals respectively. Normalizing the signals with the subject specific MVC provided a relative measure of muscle activation and force that made intra-subject comparison possible. In the case of the force-based control interface, upward forces where normalized using the mean measured force during the MVC of the biceps and downward forces were normalized using the mean measured force during the MVC of the triceps. The joystick signal was normalized to its maximum output.

For the tracking task, the velocity of the cursor was set to zero if the EMG or force signals were below a threshold of 2.5% of their MVC. This dead zone prevented that measurement noise could move the cursor. No threshold was required for the joystick control interface.

The sign of the force and joystick signals were used to determine the direction of the cursor’s movement. In the case of the EMG-based control interface the channel that presented the highest amplitude was used to control the cursor, i.e. when the biceps muscle was most active the cursor moved up and when the triceps muscle was most active the cursor moved down.

After all the aforementioned signal processing, to ensure appropriate velocity control of the cursor and to prevent fatigue, the EMG and force signals were amplified by a fixed gain that ensured that the subjects had to produce a maximum of 25% of their MVC at the peak velocity of the target in order to follow it. In the case of the joystick-based interface the angle signal was amplified with a fixed gain that resulted in the same cursor velocity at the maximum joystick angle as the EMG or force signals at 25% of their MVC.

### Data analysis

The control interfaces were evaluated analyzing the characteristics of the closed-loop system, which can be approximated by a linear transfer function (Figure 2). These characteristics will vary according to the operator’s ability to adapt to the dynamics of the controlled elements, influencing the stability and performance of the entire closed-loop system. The time records of the target (*w*(*t*)), cursor (*x*(*t*)) and error (*e*(*t*)) position signals along time, and the control signal produced by the human-interface system (*u*(*t*)) were used to evaluate the performance of the three control interfaces. First, the time records (*w*(*t*), *x*(*t*), *e*(*t*), *u*(*t*)) were transformed to the frequency domain (*W*(*f*), *X*(*f*), *E*(*f*), *U*(*f*)) via a fast Fourier transform (FFT) function and were used to estimate the power spectrums:

(1) $$\begin{array}{cc} {Ŝ}_{\text{wx}}(\;f) & =W^{*}(\;f)X(\;f)\\ {Ŝ}_{\text{ww}}(\;f) & =W^{*}(\;f)W(\;f)\\ {Ŝ}_{\text{xx}}(\;f) & =X^{*}(\;f)X(\;f)\\ {Ŝ}_{\text{eu}}(\;f) & =E^{*}(\;f)U(\;f)\\ {Ŝ}_{\text{wu}}(\;f) & =W^{*}(\;f)U(\;f) \end{array}$$

where Ŝ denotes the estimated power spectrums (the hat denotes estimate) and the asterisk (*) denotes the complex conjugate. With an observation time of 30 seconds the resultant frequency resolution is *Δω*=0.0333 Hz. Note that the time records (*w(t)*, *x(t)*, *e(t)*, *u(t)*), which lasted 180 seconds for the evaluation trials, were averaged over each subsequent block of 30 seconds for a total of 6 times in order to reduce the variance while maintaining sufficient frequency resolution.

The FRFs (${Ĥ}_{\text{xy}}$; eq. 2) and the coherence functions (${\hat{\gamma }}_{\text{wx}}^{2}$; eq. 3) of the closed-loop system were estimated only for the 10 frequencies of the multisine signal (*f*_*k*_;*k* = 1,…,10), which is known to ensure unbiased spectral estimators and relatively low variances [29].

(2) $$\begin{array}{lll} {Ĥ}_{\text{wx}}\left(\;f_{k}\right) & =\frac{{Ŝ}_{\text{wx}}\left(\;f_{k}\right)}{{Ŝ}_{\text{ww}}\left(\;f_{k}\right)},\; & \;\\ \text{where}\;\;f_{k} & =\left[0.100\;\;0.134\;\;0.200\;\;0.300\;\;0.467\;\;0.667\;\;\right)\; & \;\\ & \;\left(0.967\;\;1.4\;\;2.067\;\;3.00\right]\;\;\text{Hz}.\; \end{array}$$

(3) $${\hat{\gamma }}_{\text{wx}}^{2}\left(\;f_{k}\right)=\frac{{\left|{Ŝ}_{\text{wx}}\left(\;f_{k}\right)\right|}^{2}}{{Ŝ}_{\text{ww}}\left(\;f_{k}\right){Ŝ}_{\text{xx}}\left(\;f_{k}\right)}.$$

The coherence function is a measure of the signal to noise ratio and thus of the linearity of the dynamic process. By definition, this function equals one when there is no non-linearity and no time-varying behavior, and zero in the opposite case. These procedures used to estimate the FRFs and the coherence functions are common within system identification theory [29].

Four performance descriptors were chosen to evaluate the control interfaces: tracking error, gain margin crossover frequency, information transmission rate and effort. Furthermore, a model of the human-interface system was fitted to its estimated frequency response functions to evaluate the delay and gain parameters of each control interface.

#### *Tracking error*

The tracking error was calculated as the area under the power spectrum of the error signal (${\hat{F}}_{\text{ee}}$) from 0 to 3 Hz using the following equation:

(4) $$\begin{array}{c} {\hat{F}}_{\text{ee}}=\overset{n}{\underset{i=1}{\sum }}{Ŝ}_{\text{ee}}\left(\;f_{i}\right)\Delta \omega ,\;\;\text{where}\;\;n=\frac{f_{\text{max}}}{\text{NT}}\;\;\\ \;\text{and}\;\;{Ŝ}_{\text{ee}}\left(\;f_{i}\right)={Ŝ}_{\text{ww}}\left(\;f_{i}\right)-{Ŝ}_{\text{xx}}\left(\;f_{i}\right). \end{array}$$

*N* is the number of samples, *T* is the sampling time, *Δω* is the frequency resolution and *f*_*m* *a* *x*_ is the maximum frequency for which the tracking error was calculated (i.e. 3 Hz). This method of calculating the tracking error in the frequency domain is equivalent to the common mean squared difference between the cursor and target position signals along time [23]. A high value of *F*_*e* *e*_ indicates that the frequency content of the target and the cursor signals are different, while a low value of *F*_*e* *e*_ indicates that the frequency content of the target and the cursor signals are similar. This tracking error measure was also used to analyze the learning characteristics during the training trials.

#### *Information transmission rate*

The information transmission rate (eq. 5) quantifies the amount of information that is contained in the output signal of a communication channel, relative to the input signal [30]. In this type of studies the human-interface system can be conceived as a communication channel where the human has to transmit a movement intention through the interface [31]. We estimated the information transmission rate (Î; eq. 6) of the human-interface system for each evaluation trial by integrating Shannon’s channel capacity over the disturbed frequencies (*f*_*k*_; eq. 5). The same method to calculate the information transmission rate was used in [23,31-33].

(5) $$I=\int \log_{2}\left(1+\frac{S(\;f)}{N(\;f)}\right)\text{df}$$

(6) $$\begin{array}{lll} \hat{I} & =\frac{1}{\text{NT}}\underset{k}{\sum }\log_{2}\left(\frac{{Ŝ}_{\text{xx}}\left(\;f_{k}\right)}{{Ŝ}_{\text{xx}}\left(\;f_{k}\right)-{\left|{Ĥ}_{\text{wx}}\left(\;f_{k}\right)\right|}^{2}{Ŝ}_{\text{ww}}\left(\;f_{k}\right)}\right),\; & \;\\ & \;\;\text{where}\;\;\frac{{Ŝ}_{\text{xx}}\;\left(\;f_{k}\right)}{{Ŝ}_{\text{xx}}\;\left(\;f_{k}\right)\;-\;{\left|{Ĥ}_{\text{wx}}\;\left(\;f_{k}\right)\right|}^{2}{Ŝ}_{\text{ww}}\;\left(\;f_{k}\right)}=1\;+\;\frac{S\;\left(\;f_{k}\right)}{N\;\left(\;f_{k}\right)}.\; \end{array}$$

#### *Gain margin crossover frequency*

The gain margin crossover frequency indicates the maximum frequency at which the human can properly track the target. The gain margin crossover frequency was defined as the first frequency where the estimated phase of ${Ĥ}_{\text{wx}}$ dropped below -180 degrees. This parameter is commonly used in control engineering to analyze the stability margin of closed-loop systems.

#### *Effort measure*

The root mean square (RMS) of the velocity signal (*u*) was used to compare the required average level of velocity input during the control task between interfaces. The RMS was calculated for each period of the multisine signal, which had a duration of 30 seconds. The RMS value was interpreted as a measure of effort; assuming that when the subject produced less EMG, force or joystick movements, to complete the tracking task, the effort was lower. The increase in RMS of EMG in relation to the level of effort has been reported in several studies [34,35]. Note that the measure of effort in the case of the joystick-based interface cannot be compared to the EMG- and force-based interfaces in terms of actual physical effort as the effort required to move the joystick is not comparable to the one needed to produce the equivalent control signal using the EMG or the force interface. Nevertheless, it is still relevant to analyze with which of the control interfaces the subjects were able to produce a control signal closest to the ideal control signal needed to complete the tracking task.

#### *Learning characteristics*

The learning characteristics were analyzed calculating the tracking error for each training trial. An exponential function was fitted to the mean tracking error values as a function of trial number. We selected the first training trial as a reference to identify significant reduction of the tracking error. A performance plateau was identified when no significant reduction of the tracking error was found in all subsequent trials.

#### *Human-interface model*

To model the human-interface system (*H*_*e* *u*_) we implemented the McRuer Crossover Model [26], which is a mathematical function that describes the human controller capacities in terms of gains and time delays. According to the classic work of McRuer, during a velocity-controlled task (meaning that the plant is a first order system) the human-interface system (*H*_*e* *u*_) can be described with the following equation:

(7) $$H_{\text{mod}}(s,p)=ke^{-\mathrm{\tau s}}$$

where *k* and *τ* represent a gain and a delay respectively, *s* is the Laplace transform variable and *p* is the parameter vector *p*=[*k*, *τ*]. The values of *p* were estimated for each subject and interface from the FRF of the human-interface system by solving a non-linear least squares optimization problem using the following error cost function:

(8) $$\begin{array}{c} E(p)=\underset{k}{\sum }{\hat{\gamma }}_{\text{wx}}^{2}\left(\;f_{k}\right){\left|\ln\left(\frac{{Ĥ}_{\text{eu}}\left(\;f_{k}\right)}{H_{\text{mod}}\left(\;f_{k},p\right)}\right)\right|}^{2},\;\;\\ \;\text{where}\;\;{Ĥ}_{\text{eu}}\left(\;f_{k}\right)=\frac{{Ŝ}_{\text{wu}}\left(\;f_{k}\right)}{{Ŝ}_{\text{eu}}\left(\;f_{k}\right)}. \end{array}$$

This cost function, which has been previously used in [36,37], compares the FRFs of *H*_*m* *o* *d*_ with *H*_*e* *u*_ in order to find the gain and delay parameters that give the lowest error. Note that by using the logarithm of the FRFs we are compensating for the gain variations over the frequency spectrum [38]. Additionally, the model errors are weighted with the coherence to reduce emphasis on less reliable frequencies of the FRFs.

The fidelity of the model fit of each human-interface system was evaluated calculating the variance accounted for (VAF; eq. 9) in the time domain using the mean estimated parameters of each interface.

(9) $$\text{VAF}=\left(1-\frac{\text{var}\left(ŷ-y\right)}{\text{var}(y)}\right)100\mathrm{\%.}$$

where var(*i*) indicates variance of *i*, *y* indicates the measured output, and ŷ indicates the simulated output using the model.

#### *Statistical analysis*

We carried out a two-way repeated measures analysis of variance (RMANOVA) for each performance measure, defining the interface and the order in which the control interfaces were tested as fixed factors. Statistical test were performed with IBM SPSS software (IBM Corp. Released 2012. IBM SPSS Statistics for Windows, Version 21.0. Armonk, NY).

The testing order was not significant for any of the performance descriptors (*p* >0.78) suggesting that the training protocol was effective and cross-over learning effects were not present. The potential influence of the order was further investigated with a correlation analysis between EMG and force signals during EMG and force tasks. The correlation coefficients showed a mean value of 23% (±10% SD), which suggested that the EMG and force tasks were considerably different and therefore the order in which the subjects tested the interfaces could not introduce a significant bias to the interface performance evaluation.

Since the order did not show significant influence on the evaluation, one-way RMANOVAs were performed for each performance measure. We used *α*=0.05 (probability of Type I error) as the level of significance. A Bonferroni test was applied for pairwise comparisons.

The learning characteristics where tested with a one-way RMANOVA where each training trial was defined as a fixed factor. The influence of the order was tested for the first training trial in a similar way as in the performance evaluation and did not show any significant differences. A Sidak test was applied for pairwise comparisons as the number of fixed factors was high (i.e. 10) for this test.

## Results

**Figure 4:**
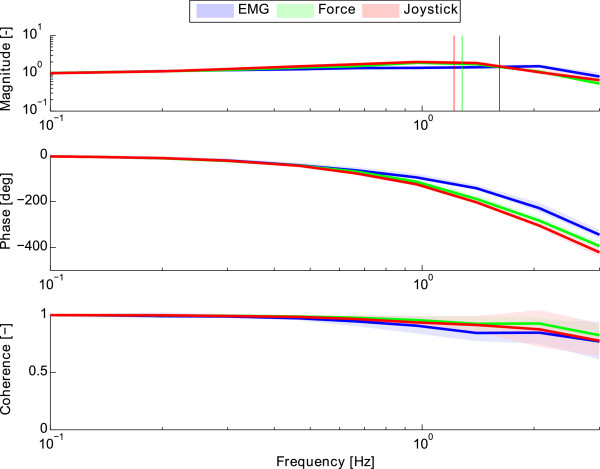
**Estimated frequency response and coherence functions of the closed-loop system (*H***_***wx***_**).** From top: magnitude, phase and coherence functions of the EMG- (blue), force- (green) and joystick-based (red) control interfaces, all as function of frequency. The solid lines indicate the mean values and the area in faded colors indicate ±1 SD. The vertical lines in the magnitude plot indicate the mean estimated gain margin crossover frequencies of each interface.

The estimated FRFs and coherence values of the closed-loop system (*H*_*w* *x*_) for each interface are shown in Figure 4. The estimated coherence values are high (${\hat{\gamma }}_{\text{wx}}^{2}>0.8$) for all three interfaces, meaning that the estimated FRFs are reliable and that the relationship between input and output is linear.

### Performance evaluation

**Figure 5:**
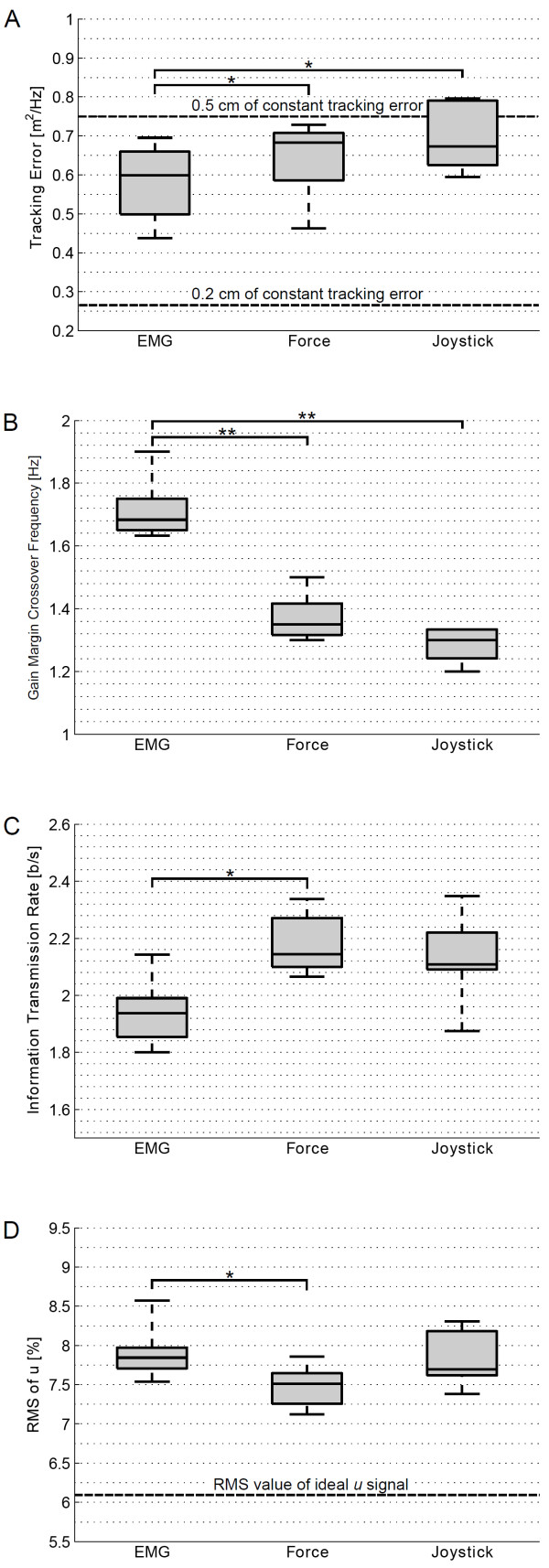
**Performance evaluation.(A)** Boxplots of the tracking error for each interface. The dashed horizontal lines indicate reference values of the tracking error. **(B)** Boxplots of the gain margin crossover frequency for each interface. **(C)** Boxplots of the information transmission rate for each interface. **(D)** Boxplots of the RMS of the velocity signal for each interface. The dashed horizontal line indicates the RMS of the optimal *u* signal. Stars indicate statistically significant differences. (*) indicates *p* <0.05, (**) indicates *p* <0.001.

All the performance descriptors presented significant differences for the RMANOVA test. However, not all pairwise comparisons between interfaces were significant (Figure 5). The EMG-based interface presented significantly lower tracking error (*p* <0.05) compared to force- and joystick-based interfaces (Figure 5A). Furthermore, the EMG-based control interface showed a significantly higher gain margin crossover frequency (*p* <0.001) than the force- and the joystick-based interfaces (Figure 5B). We also found that force-based interface provided significantly higher information transmission rates (*p* <0.05) than the EMG-based interface (Figure 5C). Finally, we found that the RMS values of the control signal *u* were significantly lower (*p* <0.05) for the force-based interface compared to the ones obtained with the EMG-based interface (Figure 5D).

**Figure 6:**
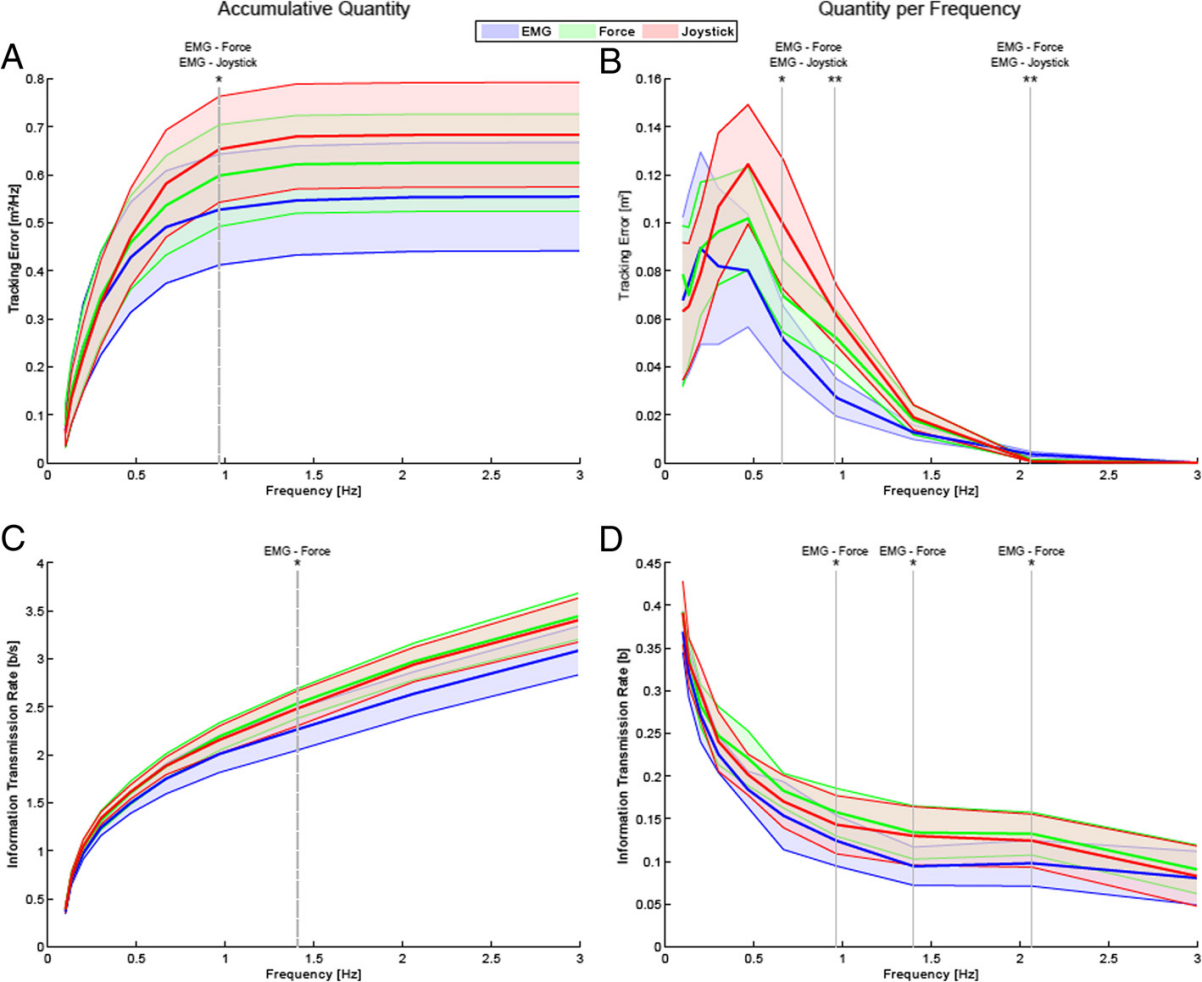
**Tracking error and information transmission rate as function of frequency of the EMG- (blue), force- (green) and joystick-based (red) control interfaces.(A)** Accumulative tracking error as function of frequency for each control interface. **(B)** Accumulative information transmission rate as function of frequency for each interface. **(C)** Tracking error per frequency of each control interface. **(D)** Information transmission rate per frequency for each interface. The solid lines indicate the mean values and the area in faded colors indicate ±1 SD. The dashed vertical lines indicate from which frequency the parameter presents statistically significant differences. The solid vertical lines indicate at which frequencies the parameter present statistically significant differences. Stars indicate statistically significant differences. (*) indicates *p* <0.05 and (**) indicates *p* <0.001. The text on top of the vertical lines indicate between which of the interfaces the differences were statistically significant.

Figure 6 shows the tracking error and the information transmission rate as function of frequency measured accumulatively and per frequency. Note that the progression of these quantities as function of frequency is affected by the fact that the multisine signal used as input (*w*) presented larger power at low frequencies. As a result the tracking error and the information transmission rate presents larger magnitudes at low frequencies when measured per frequency, and they rise quickly at low frequencies when measured accumulatively. We emphasize that the aim of Figure 6 is not to provide a relative comparison of the quantities along the frequency spectrum but to compare the quantities between the three interfaces for specific frequencies. The accumulative tracking error of the EMG-based interface becomes significantly lower compared to the force- and joystick-based interfaces beyond 0.9 Hz (Figure 6A). The accumulative information transmission rate of the EMG-based interface becomes significantly lower (*p* <0.05) compared to the force-based interface beyond 1.4 Hz (Figure 6C). The tracking error per frequency of the EMG-based interface is significantly lower at 0.6 (*p* <0.05) and 0.9 (*p* <0.001) Hz, and significantly higher (*p* <0.001) at 2.06 Hz compared to the force- and joystick-based interfaces (Figure 6B). The information transmission rate per frequency of the EMG-based interface is significantly lower (*p* <0.05) at 0.9, 1.4 and 2.06 Hz compared to the force-based interface (Figure 6D).

### Learning characteristics

**Figure 7:**
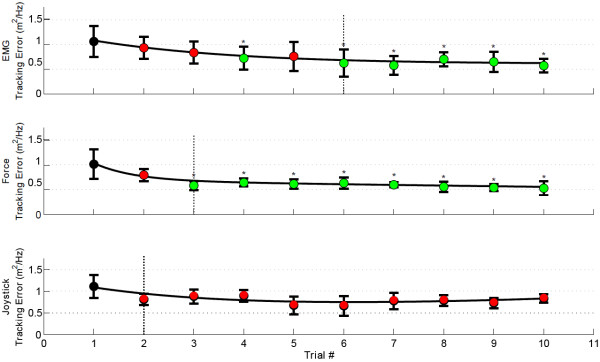
**Learning curves.** Tracking error along the ten training trials for the EMG-, force- and joystick-based control interfaces. An exponential function was fitted to the mean tracking error of each training trial. The first training trial was used as a reference to identify significant reductions of tracking error. The green markers indicate significant reduction of tracking error (*p* <0.05) relative to the first trial. The red markers indicate non-significant reduction of tracking error (*p* >0.05) relative to the first training trial. The vertical lines indicate the trial in which the performance plateau was identified. The error bars indicate ± 1 SD. Stars indicate statistically significant differences. (*) indicates *p* <0.05.

Figure 7 shows the learning curves obtained from fitting an exponential function to the mean values of the tracking error of each training trial. For the EMG-based control interface there was a significant reduction of tracking error (*p* <0.05) relative to the first training trial at the 6^*t* *h*^ trial, while the force-based interface presented a significant reduction (*p* <0.05) in the 3^*r* *d*^ trial. The joystick-based interface did not show any significant reduction of the tracking error. The learning curves also show that all interfaces reached a performance plateau before the end of the training.

### Human-interface model

**Figure 8:**
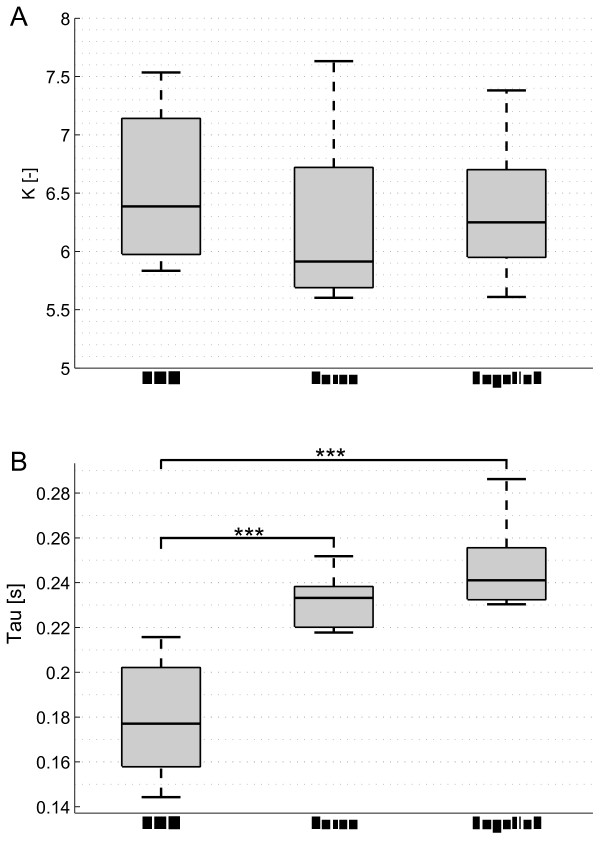
**Estimated parameters of the human-interface model (**${Ĥ}_{\text{eu}}$**).(A)** Boxplots of the gain parameter for each interface. **(B)** Boxplot of the delay parameter for each interface. Stars indicate statistically significance differences. (***) indicates *p* <0.001.

The results of the parameter estimation of k and *τ* are shown in Figure 8. We found a VAF measure of 98.8%, 96.7% and 82.9% for the EMG-, force- and joystick- based interfaces respectively. The high VAF values indicate that the observed behavior is well described by the model within the measured frequency range. While we did not find a significant difference between the estimated gain parameters (*k*), the EMG-based interface presented significantly lower delay (*p* <0.001) than the force- and the joystick-based interfaces.

### Participant’s opinion

The results from the questionnaire show that six out of eight participants preferred the force-based interface followed by EMG- and joystick-based interfaces. The other two participants preferred EMG-based interface the most, followed by force- and joystick-based interfaces.

## Discussion

The amplitude range of the joystick interface was smaller compared to the other two interfaces, for which the amplitude limits were set according to the maximum force or EMG signal that the subject could generate (i.e. MVC). This very sensitive and limited range of the joystick might be the cause of the reduced user acceptance. Nevertheless, the performance of the joystick interface was similar to the force-based interface for all the performance descriptors (Figure 5). Our motivation to test a classic hand-joystick with small input range was that this type of interface is commonly used by individuals with severe muscular weakness to control electric wheelchairs, domestic devices and external robotic arms. Therefore, it makes sense to consider the option of also using this control interface to operate an active arm support. Comparing a classic hand-joystick to new interfaces (from a patient’s point of view) is especially relevant for the targeted patient group, as the performance of a new control interface needs to represent a meaningful improvement and worth the effort of learning and adaption.

In accordance with the results by Corbett et al. [23] we also found that the EMG-based interface presented a significantly lower tracking error than the force-based interface (Figure 5A). Interestingly the tracking error per frequency of the EMG-based interface becomes significantly higher at 2 Hz compared to the force- and joystick-based interface (Figure 6B). This performance change might be caused by the significant decrease of information transmission rate of the EMG-based interface beyond 1.4 Hz (Figure 6C).

Regarding the performance measure of the gain margin crossover frequency, the participants were able to track frequencies up to 1.7 Hz when using the EMG-based control interface, while they were able to track frequencies only up to 1.3 Hz with the other two interfaces (Figure 5B). From the parameters estimation of the human-interface system we can conclude that the larger gain margin crossover frequency of the EMG interface is possible due to its low delay (Figure 8B). Note that the EMG signals are measured earlier than their resultant force or motion signals, which pass through the muscle and skeleton dynamics. Despite having a higher gain margin crossover frequency, the EMG-based interface presented a significantly lower information transmission rate beyond 1.4 Hz (Figure 6C) due to its lower signal to noise ratio (see also lower coherence in Figure 4) compared to the force and joystick signals.

Figure 6C shows that, unlike found in [23], significant differences between EMG- and force-based interfaces in terms of information transmission rate appear beyond 1.4 Hz. We conjecture that the study by Corbett et al. [23] could not find equivalent significance due to the limited bandwidth (1 Hz) of the used tracking task.

The results of the effort comparison showed that the force-based interface had significantly lower RMS value of the control signal compared to the EMG- and joystick-based interfaces (Figure 5D). An analysis of the EMG data during both EMG and force tasks indicated that the difference in RMS values was caused by the higher presence of co-contraction when using EMG as control interface.

The VAF measures indicated that the parameters found for the EMG- and force-based interfaces described the human-interface system with very high fidelity. However, this was not the case for the joystick-based interface, for which the model could explain 83% of the measured data. These results suggest that a slightly different model should be used to describe the joystick-based interface more precisely.

With regard to the learning curves, we can observe, as expected, that as the training proceeded, the subjects learned and the tracking error became smaller indicating that the frequency content of the target and the cursor became increasingly similar. The results showed that despite the fact that the EMG-based interface is far from the natural means to interact with the environment while the force-based interface is closer, the difference in terms of learning cost was small: the participants were able to reach a performance plateau with the EMG-based interface after the 6^*th*^ training trial, which is only three trials after the same plateau was reached with the force-based interface (Figure 7). Regarding the joystick-based interface, we did not find any significant improvement of the tracking error after the 10^*th*^ training trial. The lack of a significant learning effect with the joystick could be a consequence of the prior experience of the participants with these type of devices, thus no further detectable improvement was achieved during the experiment.

## Conclusions

In the context of the wide variety of invasive and non-invasive control interfaces for active movement-assistive devices [39-41], our study characterizes and compares the performance, learning characteristics and subjective preference of EMG, force and hand joystick as control interfaces for active arm supports using a one-dimensional screen-based position-tracking task.

None of the evaluated interfaces was superior in all of the four performance descriptors that we have analyzed (Table 1). EMG-based interface was superior in terms of tracking error and gain margin crossover frequency compared to the force- and joystick-based interfaces. The force-based interface was superior in terms of information transmission rate and effort compared to the EMG-based control interface. While the results of the tracking error are in accordance with the findings reported by [23], the significant differences found in terms of information transmission rate were unnoticed in the aforementioned study probably because these are present beyond 1.4 Hz only. Strictly speaking, the joystick was always tied with the force-based interface in all four performance descriptors, although our results were close to significance for considering the joystick as the worst option in terms of gain margin crossover frequency and effort.

**Table 1:** Overview of the performance, learning and preference evaluation

	**EMG**	**Force**	**Joystick**
**Tracking Error**	1	2	2
**GM Crossover Freq.**	1	2	2
**ITR**	2	1	-
**Effort**	2	1	-
**Learning**	2	2	1
**Preference**	2	1	3

The ranking of the interfaces for each performance descriptor is indicated with numbers where 1 indicates the best interface for a specific performance descriptor. GM Crossover Freq.: gain margin crossover frequency ITR: information transmission rate.

Our modeling of the human-interface system revealed that the high gain margin crossover frequency of the EMG-based interface was related to its low delay. This finding should be especially considered in interfaces where high bandwidths are required, such in the case of empowering exoskeletons in which target bandwidths are in the order of 2 Hz [42].

Probably because of being a well-known device, with which all participants had already some experience, the joystick showed no learning effects in these experiments. The other two interfaces were not hard to learn, with the force-based interface showing the fastest learning curve. Surprisingly, despite being the most familiar interface, the joystick was the least preferred interface because participants disliked its high sensitivity over the small amplitude range.

In practice, the performance descriptors should be weighted according to the requirements of the specific application to select the most suitable interface for a particular case. In the context of our specific application of controlling an arm support for people with muscular weakness, tracking error and the effort measure should be weighted heavily. Also, criteria such as preference and learning are likely to show significant differences among patients with different pathologies.

From these results we conclude that all of the control interfaces considered in this study can be regarded as a candidate interface for the control of an active arm support in patients with muscular weakness. Our future work will have to consider testing them all using a functional task closer to an activity of daily living, (e.g. a two- or three-dimensional reaching-retrieving task). Apart from the performance criteria evaluated in this study, we will consider additional design requirements, such as ease of use, portability and comfort.

## Abbreviations

EMG: Electromyogram; DOF: Degree of freedom; MVC: Maximum voluntary contraction; FRF: Frequency response function; RMS: Root mean square; SD: Standard deviation; RMANOVA: Repeated measures analysis of variance.

## Competing interests

The authors declare that they have no competing interests.

## Authors’ contributions

All the authors have made substantial contributions to conception and design, acquisition, analysis and interpretation of data; have been involved in drafting and revising the manuscript; and have given final approval of the version to be published. All authors read and approved the final manuscript.
